# A Case of Duodenal Metastasis From a Poorly Differentiated Lung Carcinoma

**DOI:** 10.7759/cureus.115144

**Published:** 2026-08-25

**Authors:** Caroline Meurer Da Cunha, Sebastian Hernandez Mejia, Svetoslav Bardarov

**Affiliations:** 1 Internal Medicine, Richmond University Medical Center, New York, USA; 2 Internal Medicine/Cardiology, Richmond University Medical Center, New York, USA; 3 Pathology and Laboratory Medicine, Richmond University Medical Center, New York, USA

**Keywords:** ca lung, duodenum metastasis, gastro-intestinal metastasis, primary lung carcinoma, rapid progression

## Abstract

Lung cancer is one of the leading causes of cancer-related mortality worldwide and commonly metastasizes to the brain, bones, liver, and adrenal glands, whereas gastrointestinal (GI) involvement is uncommon and rarely diagnosed during a patient’s lifetime. We report a rare case of poorly differentiated carcinoma of the lung with duodenal metastasis. The patient was a 73-year-old man who presented with abdominal pain and melena, prompting esophagogastroduodenoscopy (EGD), which revealed ulcerated duodenal lesions. Histopathologic examination of the duodenal biopsy demonstrated a poorly differentiated carcinoma morphologically similar to the known lung tumor. Immunohistochemical analysis showed positivity for pancytokeratin (AE1/AE3) and PD-L1, with negative staining for TTF-1, CK7, CDX2, CK5/6, p63, neuroendocrine markers, and lymphoid markers, supporting metastatic disease when considered alongside the patient's known primary lung malignancy and clinicopathologic findings.

As the lesions were neither actively bleeding nor causing obstruction, no endoscopic or surgical intervention was required. Despite initiation of oncologic therapy, the patient’s condition rapidly deteriorated, and he died during the same hospitalization. The interval from diagnosis of the primary lung tumor to identification of GI involvement was only two months, followed by a further survival of 28 days after identification of duodenal involvement. This report underscores the importance of maintaining a high index of suspicion for GI metastases in patients with lung cancer who develop GI symptoms, as timely recognition may facilitate appropriate diagnostic evaluation and management.

## Introduction

Lung cancer remains the leading cause of cancer-related mortality worldwide among both men and women. In the United States, lung cancer accounts for nearly 2.5 times as many deaths as colorectal and pancreatic cancers, the second- and third-leading causes, respectively [1]. Lung cancers are broadly classified into two major categories: non-small cell lung cancer (NSCLC), which accounts for the majority of cases and includes adenocarcinoma, squamous cell carcinoma, and other less common histologic subtypes, and small cell lung cancer (SCLC), a high-grade neuroendocrine carcinoma characterized by its aggressive behavior and early metastatic spread.

Due to their often silent progression, high propensity for metastasis, and rapid dissemination, lung cancers are frequently diagnosed at an advanced stage, by which time metastatic disease has already occurred [2]. The most common sites of metastasis include the brain, bones, liver, adrenal glands, and lymph nodes, particularly the mediastinal and supraclavicular lymph nodes. However, metastases to less typical locations, such as skeletal muscle, skin, tongue, and gastrointestinal (GI) tract, have also been reported [3,4]. The metastatic process is multifactorial and involves several key processes, including epithelial-mesenchymal transition (EMT), vascular and lymphatic invasion, and the establishment of pre-metastatic niches in distant organs [5]. These processes allow malignant cells to detach from the primary tumor, survive in the circulation, and colonize distant tissues.

Although GI involvement is rare, metastases from primary lung cancer have been documented in organs such as the tongue, esophagus, stomach, pancreas, small intestine, and colon [6]. The clinical presentation varies depending on the site and extent of the lesion. Patients may be asymptomatic or present with dysphagia, early satiety, anemia suggestive of upper or lower GI bleeding, abdominal pain or distension, and changes in bowel habits. Despite their rarity, GI metastases from lung carcinoma should be considered in patients with known or suspected pulmonary malignancy who present with nonspecific GI symptoms or unexplained lesions. Because these metastases are frequently not recognized during life and may mimic primary GI malignancies, they pose a significant diagnostic challenge. Prompt recognition of metastatic disease is important for appropriate diagnostic evaluation and clinical management. We describe a case of poorly differentiated carcinoma of the lung, without definitive squamous or adenocarcinoma differentiation, presenting with duodenal metastasis, highlighting the importance of maintaining a high index of clinical suspicion for atypical metastatic patterns.

## Case presentation

**Figure 1:**
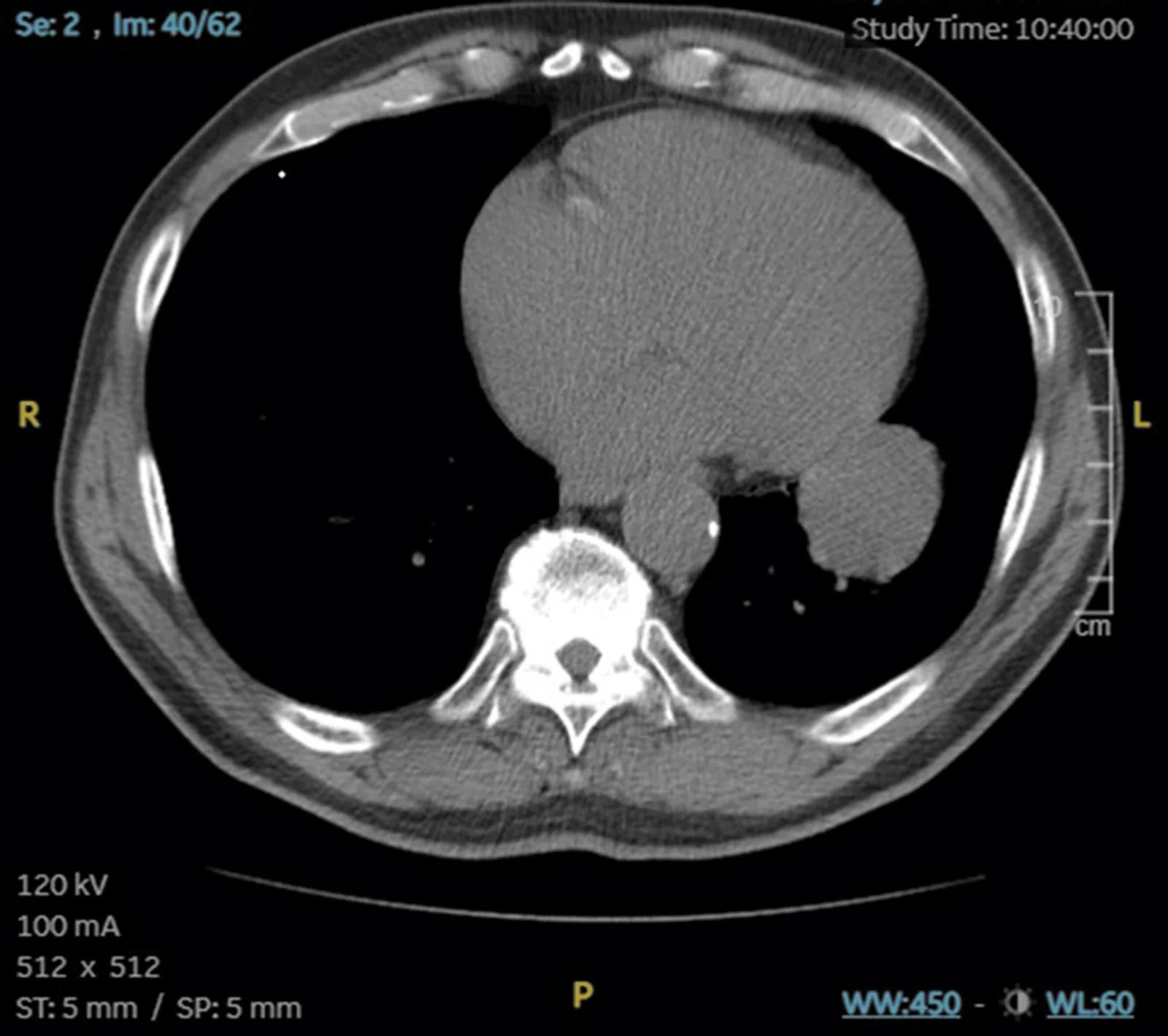
Non-contrast CT chest demonstrating a new left lower lobe pulmonary mass with soft-tissue attenuation Axial non-contrast CT of the chest demonstrates a newly identified left lower lobe pulmonary mass measuring 4.5 × 5.3 × 5.0 cm. The lesion demonstrates soft-tissue attenuation with an average density of approximately 36 HU on soft-tissue window imaging CT: computed tomography

**Figure 2:**
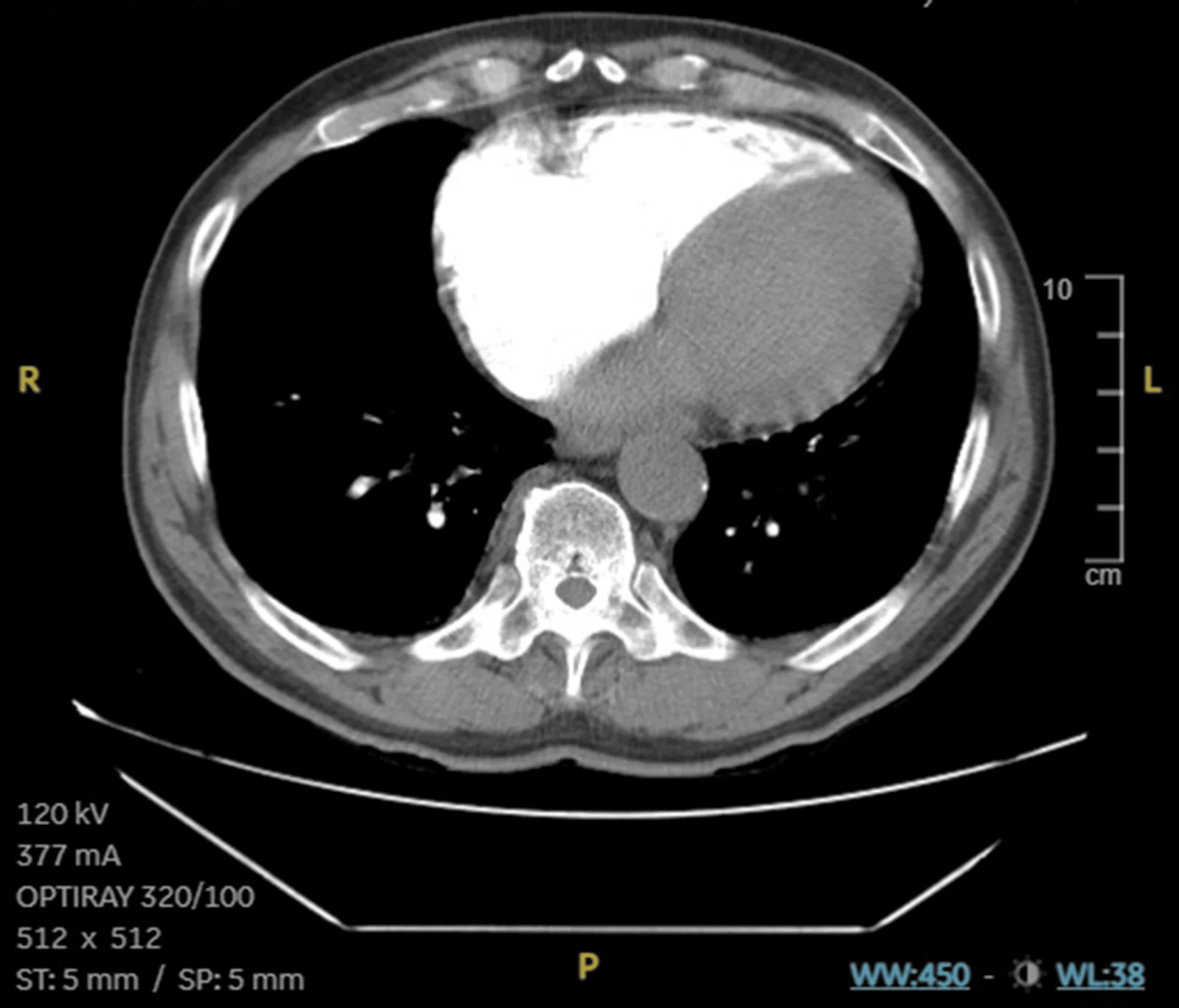
Prior contrast-enhanced CT chest demonstrating absence of the left lower lobe pulmonary mass Axial contrast-enhanced CT of the chest obtained eight months before presentation demonstrates emphysematous changes without evidence of a left lower lobe pulmonary mass. This prior study provides comparison with the subsequent development of the newly identified left lower lobe lesion CT: computed tomography

In September 2025, a 73-year-old man with a history of heart failure with preserved ejection fraction (HFpEF), coronary artery disease (CAD), and a more than 30 pack-year smoking history was incidentally found to have a left lower lobe lung mass on a CT abdomen performed for the evaluation of abdominal pain. A dedicated chest CT (Figure 1) confirmed a new, rapidly progressive left lower lobe mass measuring 4.5 × 5.3 × 5 cm, with soft-tissue attenuation of approximately 36 Hounsfield units (HU). For comparison, prior imaging from 2024 showed mild emphysematous changes but no lung mass at the time (Figure 2).

**Figure 3:**
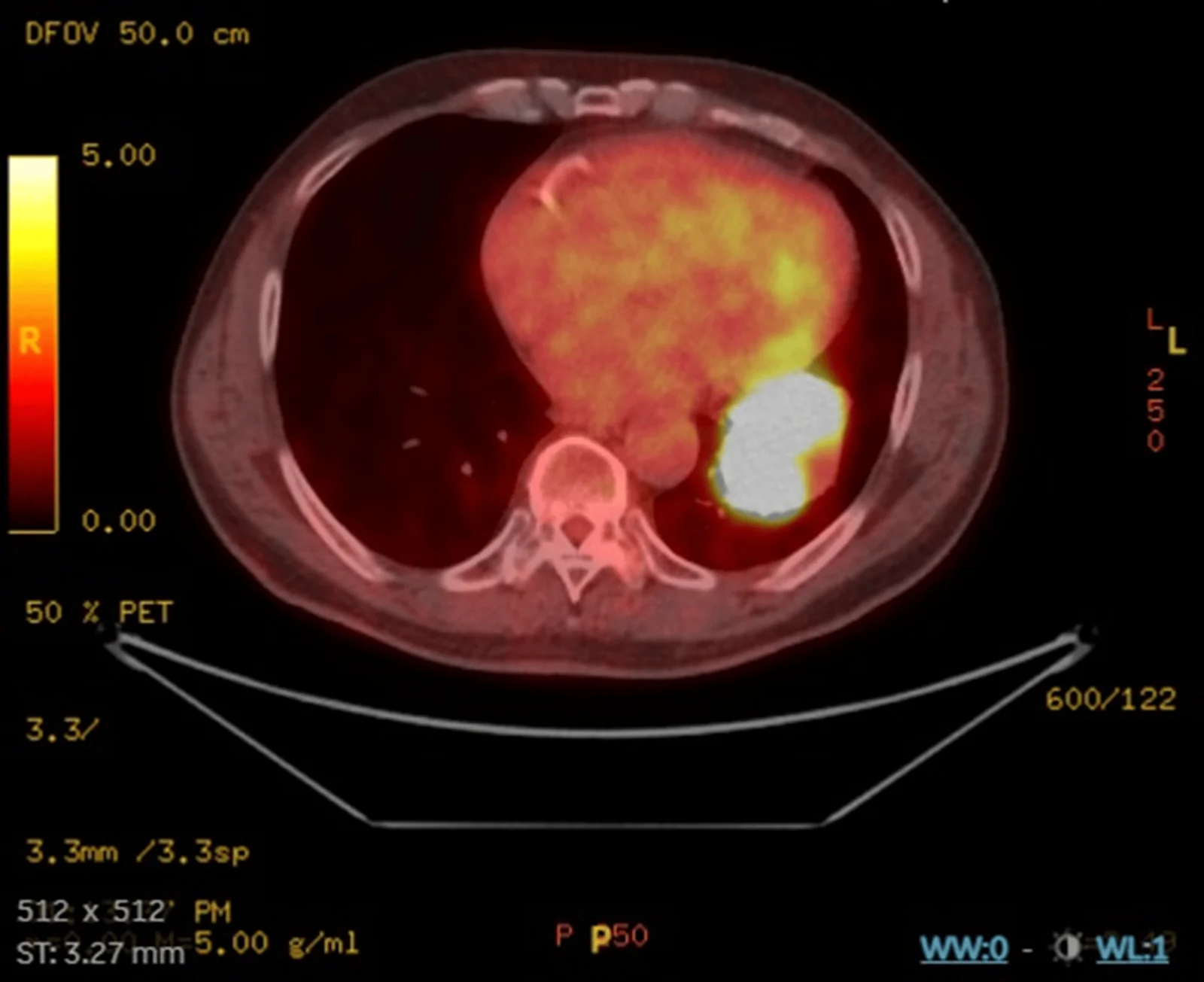
PET/CT demonstrating intense FDG uptake within the left lower lobe pulmonary mass Whole-body FDG PET/CT from the skull base to the mid-thigh demonstrates intense FDG uptake within a 5.5 cm left lower lobe pulmonary mass, consistent with hypermetabolic activity. Axial PET/CT imaging demonstrates the lesion’s relationship to the adjacent cardiac and mediastinal structures PET/CT: positron emission tomography/computed tomography; FDG: fluorodeoxyglucose

The patient was further referred to a pulmonologist, who subsequently obtained a PET-CT scan from the skull base to mid-thigh and performed a percutaneous biopsy of the left lung mass. Later that month, the PET-CT demonstrated increased glucose metabolic activity within a large 5.5 cm mass in the left lower lung lobe, which was highly suspicious for a primary lung malignancy, with no evidence of mediastinal lymphadenopathy or distant metastatic disease (Figure 3).

**Figure 4:**
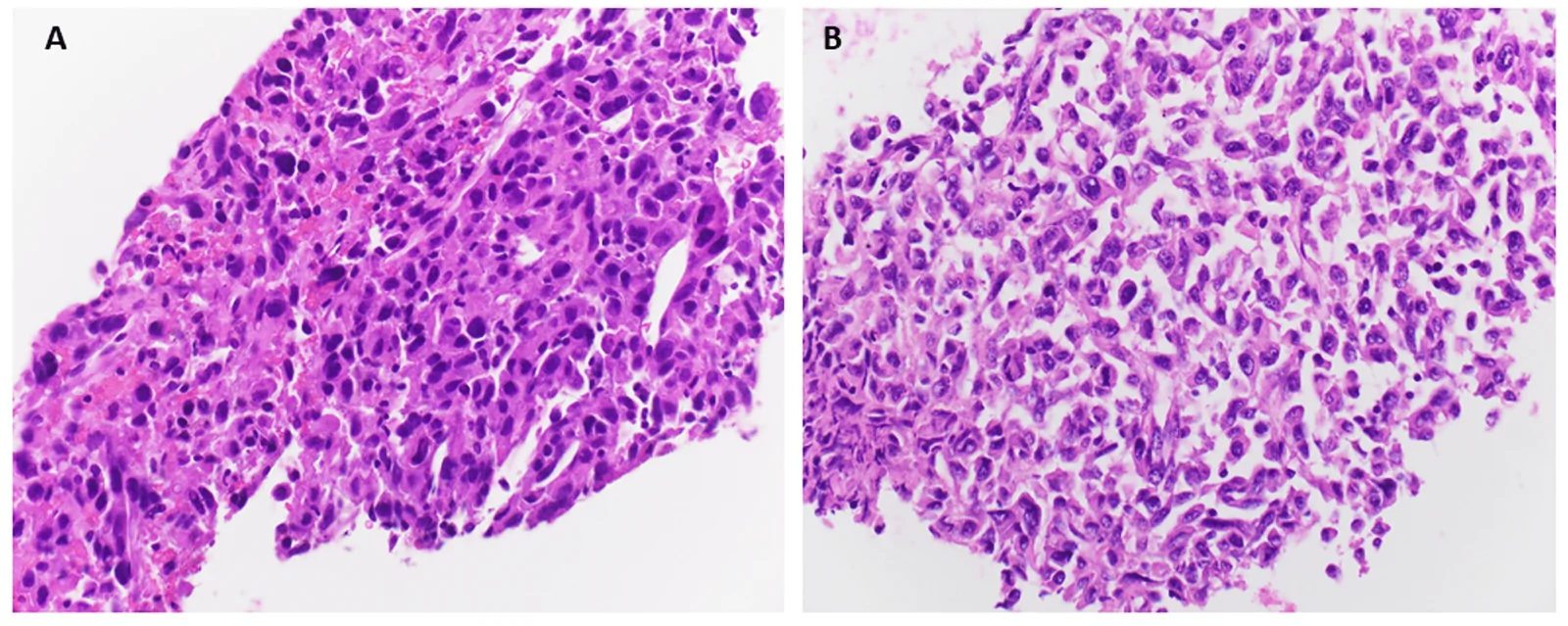
Histopathological findings of the primary lung tumor and metastatic mediastinal lymph node Histopathological examination demonstrating (A) biopsy of the left lower lobe pulmonary mass, showing a poorly differentiated carcinoma, and (B) mediastinal lymph node biopsy obtained by EBUS-TBNA, demonstrating a metastatic epithelial neoplasm morphologically identical to the primary lung tumor, confirming nodal metastasis EBUS-TBNA: endobronchial ultrasound-guided transbronchial needle aspiration

Histopathological examination of the lung mass biopsy confirmed a poorly differentiated carcinoma. Immunohistochemical analysis demonstrated that the tumor cells were positive for pancytokeratin (AE1/AE3) and negative for CK7, TTF-1, CK5/6, p63, CD56, synaptophysin, chromogranin, CD45, CD3, CD20, and S-100. Biopsy of the mediastinal lymph nodes, obtained via endobronchial ultrasound for staging purposes, revealed a metastatic epithelial neoplasm that was morphologically identical to the primary lung tumor, thereby confirming nodal metastasis (Figure 4).

The patient was referred for outpatient oncology follow-up, and further evaluation, including brain MRI and genomic profiling, was requested. Brain MRI demonstrated multiple bilateral enhancing lesions consistent with metastatic disease. Genomic profiling using the Guardant360® assay detected a KRAS G12C mutation (Table 1). KRAS alterations are recognized oncogenic drivers in lung carcinomas, and the KRAS G12C variant is clinically relevant due to the availability of targeted therapies for selected advanced lung malignancies harboring this alteration [7]. Although the histopathological and immunohistochemical findings did not allow for definitive classification of the tumor as a specific lung carcinoma subtype, this molecular finding provided additional characterization of the tumor and highlighted its potential therapeutic relevance.

**Table 1:** Genomic profiling results obtained using the Guardant360® assay Genomic alterations identified by next-generation sequencing using the Guardant360® liquid biopsy assay

Detected alterations/biomarker(s)	Variant allele fraction (VAF)
KRAS G12C	46.7%
TP53 R273L	38.5%
TERT promoter SNV	33.9%
SMARCA4 G1232V	32.9%
MSH6 E665	22.9%
CCND1 amplification	Copy number 6.2
CDKN2A biallelic loss (homozygous deletion)	Copy number 1.4
KRAS amplification	Copy number 3.3
MTAP loss	Copy number 1.4

**Figure 5:**
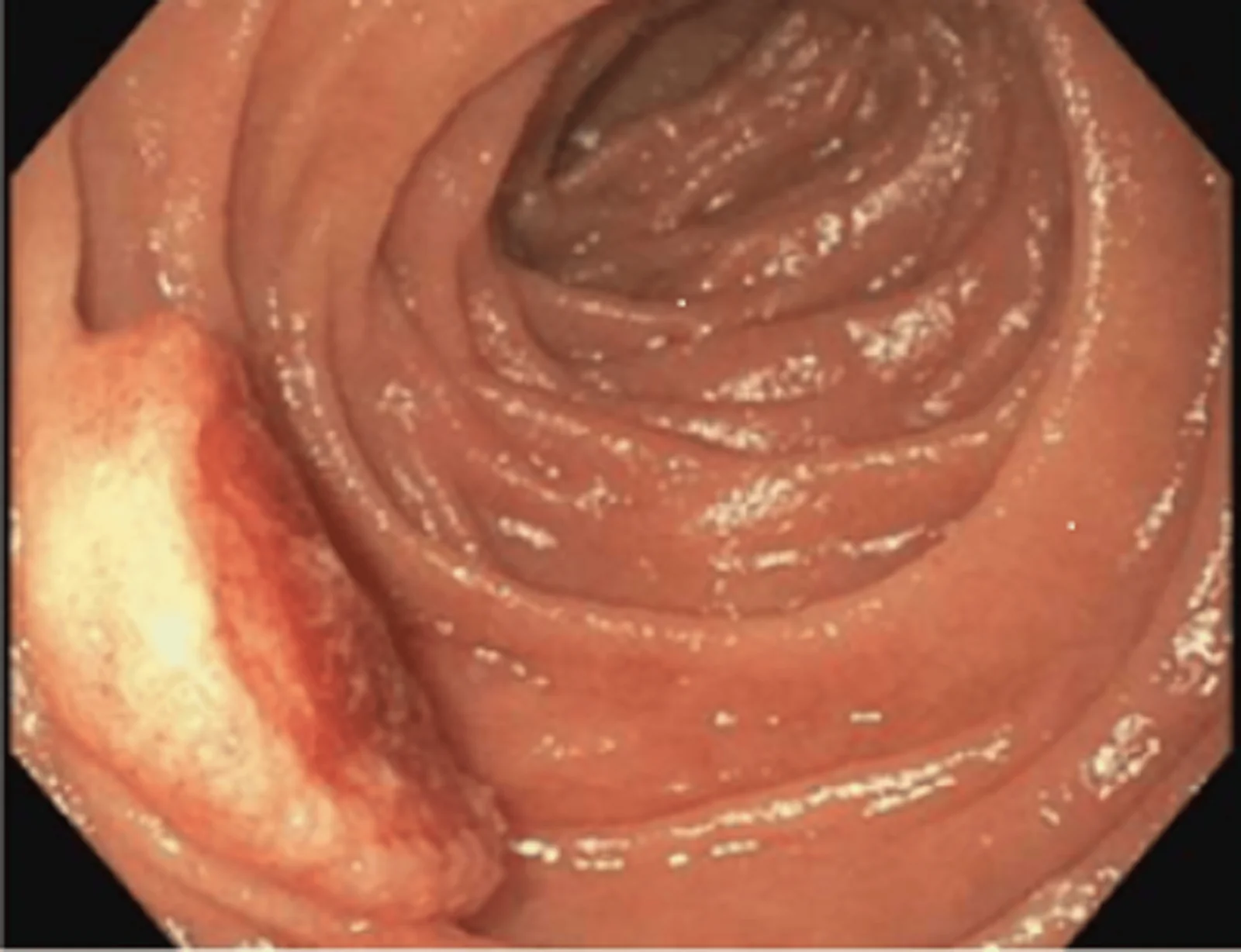
EGD demonstrating an ulcerated duodenal mass in the second portion of the duodenum EGD demonstrates a 3.5 cm ulcerated, everted mass in the second portion of the duodenum EGD: esophagogastroduodenoscopy

**Figure 6:**
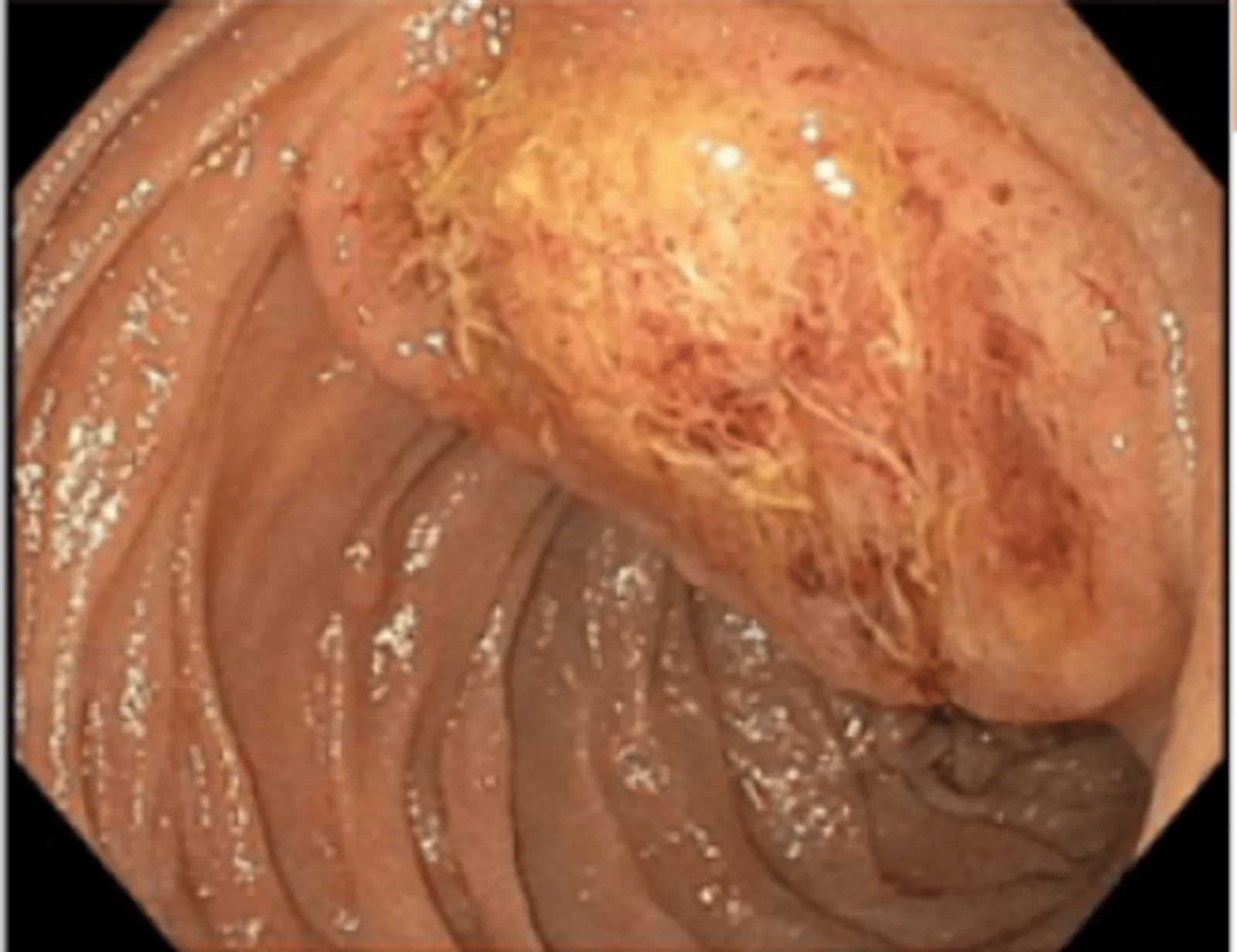
Additional endoscopic view of the ulcerated duodenal mass Additional EGD image demonstrating the ulcerated, everted mass in the second portion of the duodenum from a different endoscopic perspective EGD: esophagogastroduodenoscopy

Before the initiation of systemic therapy, the patient presented to the emergency department with a two-day history of dark, hard stools. On the day of presentation, he reported fatigue and shortness of breath and denied hematochezia or hematemesis. Given the clinical presentation, an upper gastrointestinal bleed was suspected, with gastritis and peptic ulcer disease considered in the differential diagnosis. Physical examination revealed a soft abdomen with normal bowel sounds and epigastric tenderness. Esophagogastroduodenoscopy (EGD) revealed a 3.5-cm ulcerated, everted lesion in the second portion of the duodenum without active bleeding (Figures 5, 6). Biopsy demonstrated a poorly differentiated carcinoma morphologically similar to the primary lung tumor, consistent with metastatic involvement.

**Figure 7:**
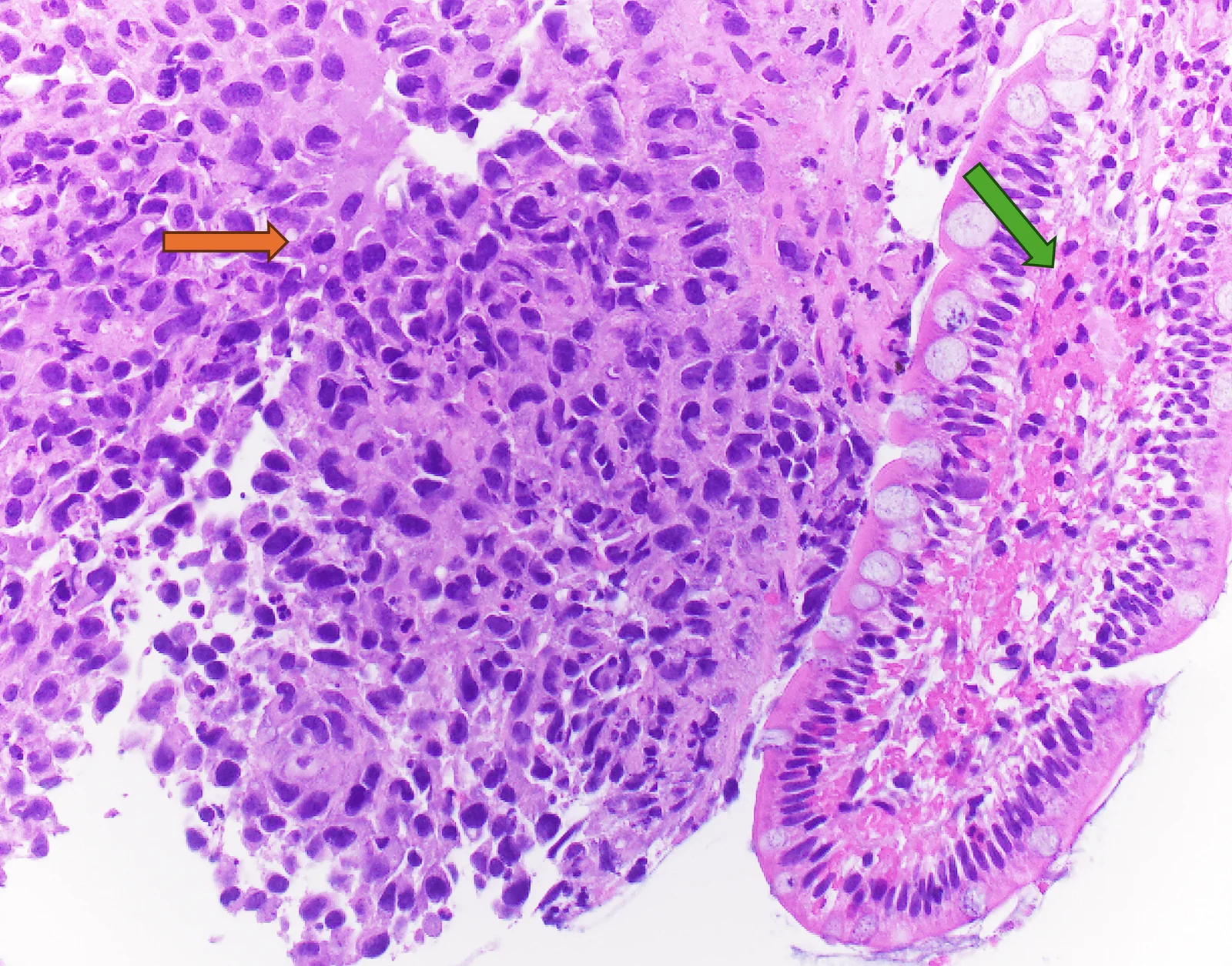
Histopathological examination of the duodenal biopsy Histopathological examination of the duodenal biopsy demonstrates neoplastic infiltration. The green arrow indicates preserved normal duodenal villi, while the orange arrow highlights the neoplastic infiltrate replacing the normal mucosal architecture

Histopathology showed an infiltrative, poorly differentiated carcinoma composed of medium-sized cells with an elevated nuclear-to-cytoplasmic (N:C) ratio, irregular nuclear membranes, prominent “cherry-red” nucleoli, and numerous mitotic figures. Immunohistochemical analysis demonstrated that the neoplastic cells were positive for pancytokeratin (AE1/AE3), supporting their epithelial origin. They were negative for CK7, CK20, CDX2, CD34, and SALL4. This profile was identical to the immunophenotype observed in the primary lung neoplasm. In addition, PD-L1 immunostaining was positive in the lesional cells. Mismatch repair (MMR) proteins were intact, with normal expression of MLH1, MSH2, MSH6, and PMS2 (Figure 7).

Comprehensive genomic profiling identified a KRAS G12C mutation with associated KRAS amplification. The mutation profile also revealed SMARCA4 G1232V, MSH6 E665*, CCND1 amplification, CDK2A biallelic loss (homozygous deletion), and MTAP loss. TP53 R273L and a TERT promoter SNV were not detected. Tumor mutational burden (TMB) was 20.93 mutations per megabase, and microsatellite status was stable (MSS). This profile suggests a tumor with multiple accumulated genomic alterations, consistent with advanced-stage disease.

**Figure 8:**
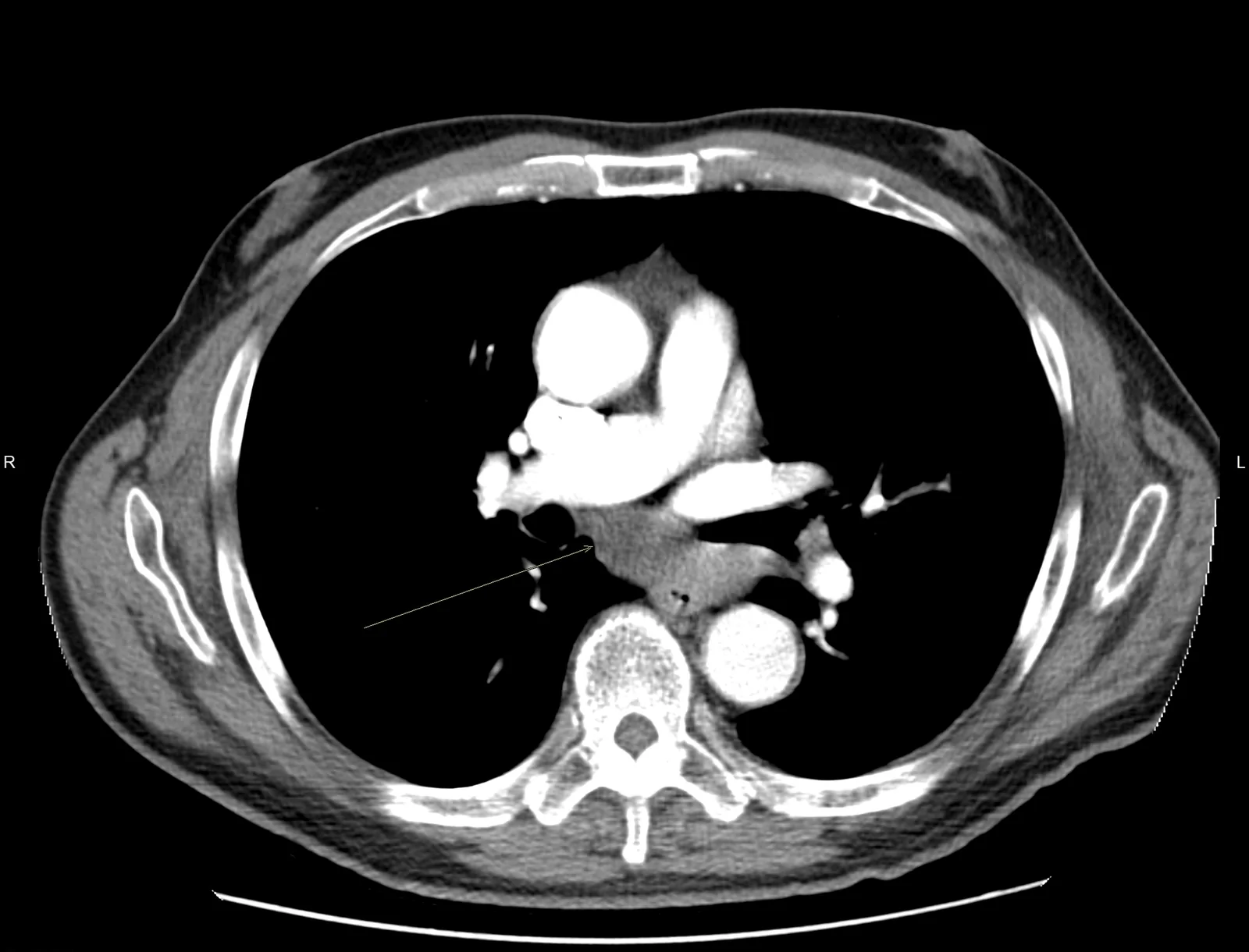
Chest CT demonstrating subcarinal lymphadenopathy Axial chest CT demonstrates subcarinal lymphadenopathy. The arrow indicates an enlarged subcarinal lymph node measuring approximately 2.4 × 3.4 cm CT: computed tomography

**Figure 9:**
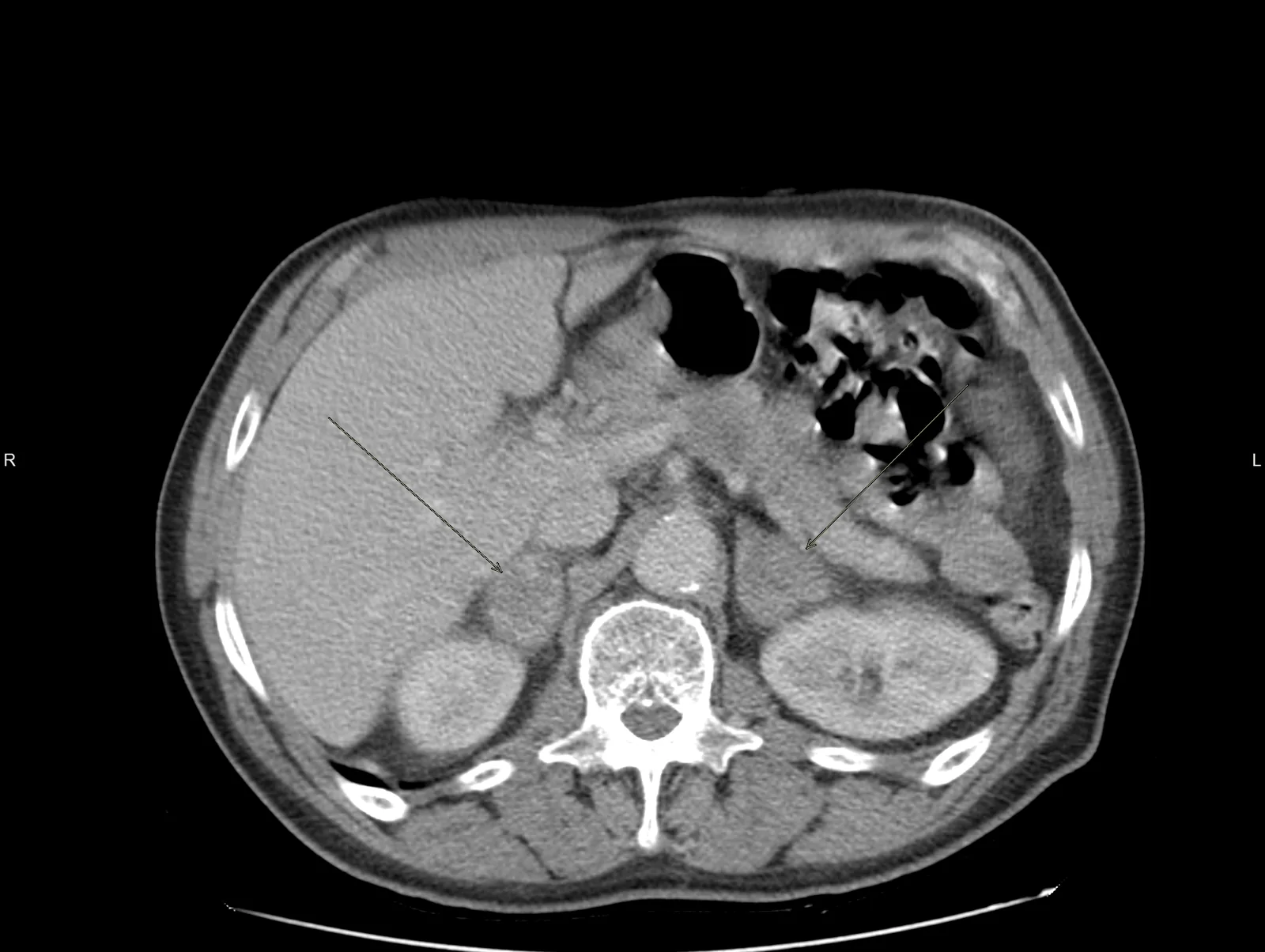
Abdominal CT demonstrating bilateral adrenal masses Axial CT of the abdomen demonstrates bilateral adrenal masses. The arrows indicate enlarged adrenal lesions measuring approximately 4.7 × 2.9 cm on the left and 3.5 × 2.5 cm on the right. Image interpretation is limited by motion artifact during image acquisition CT: computed tomography

**Figure 10:**
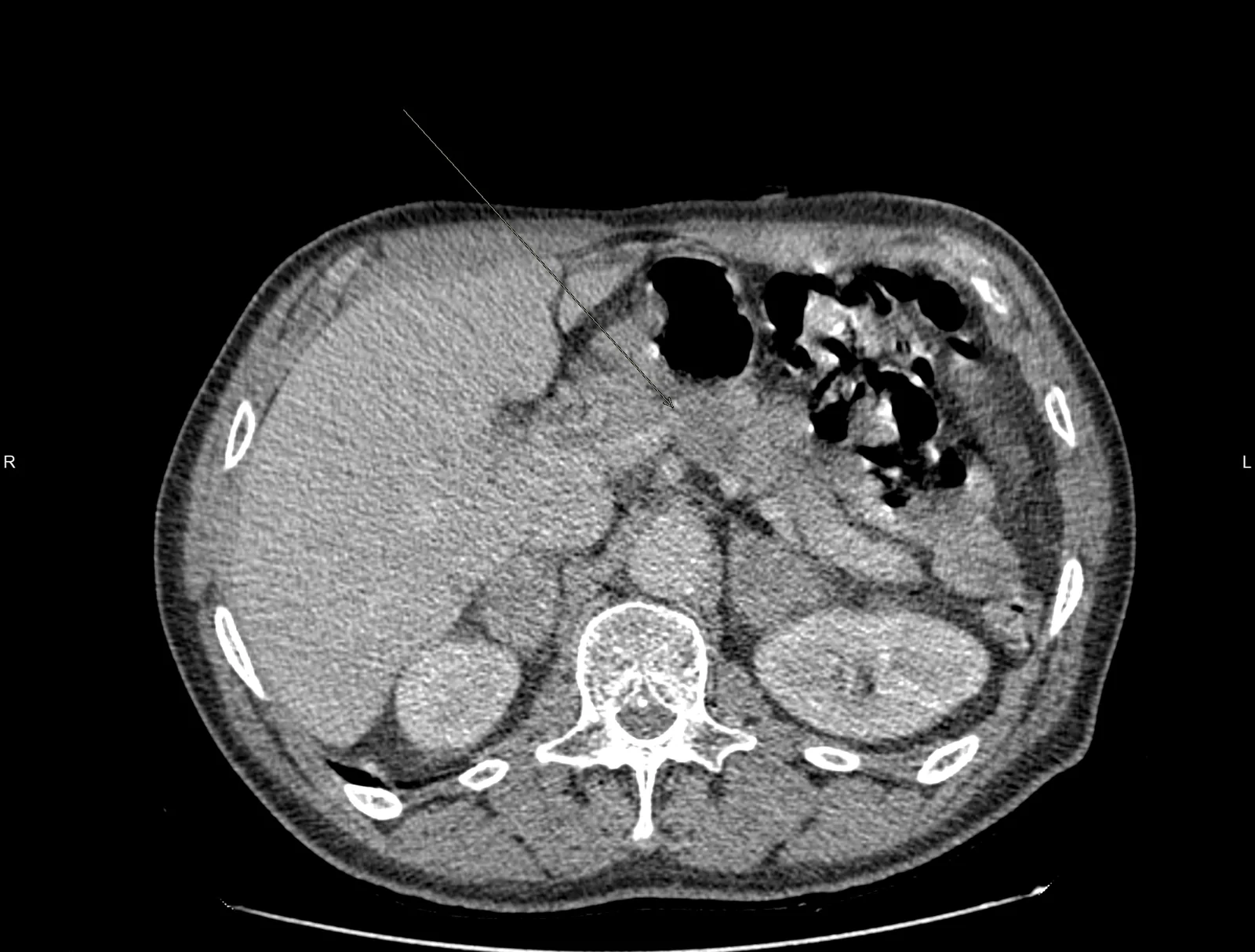
Abdominal CT demonstrating an ill-defined pancreatic body lesion Axial CT of the abdomen demonstrates an ill-defined hypodense lesion within the body of the pancreas. The arrow indicates the pancreatic lesion, measuring approximately 1.0 × 2.0 cm. Image interpretation is limited by motion artifact during image acquisition CT: computed tomography

**Figure 11:**
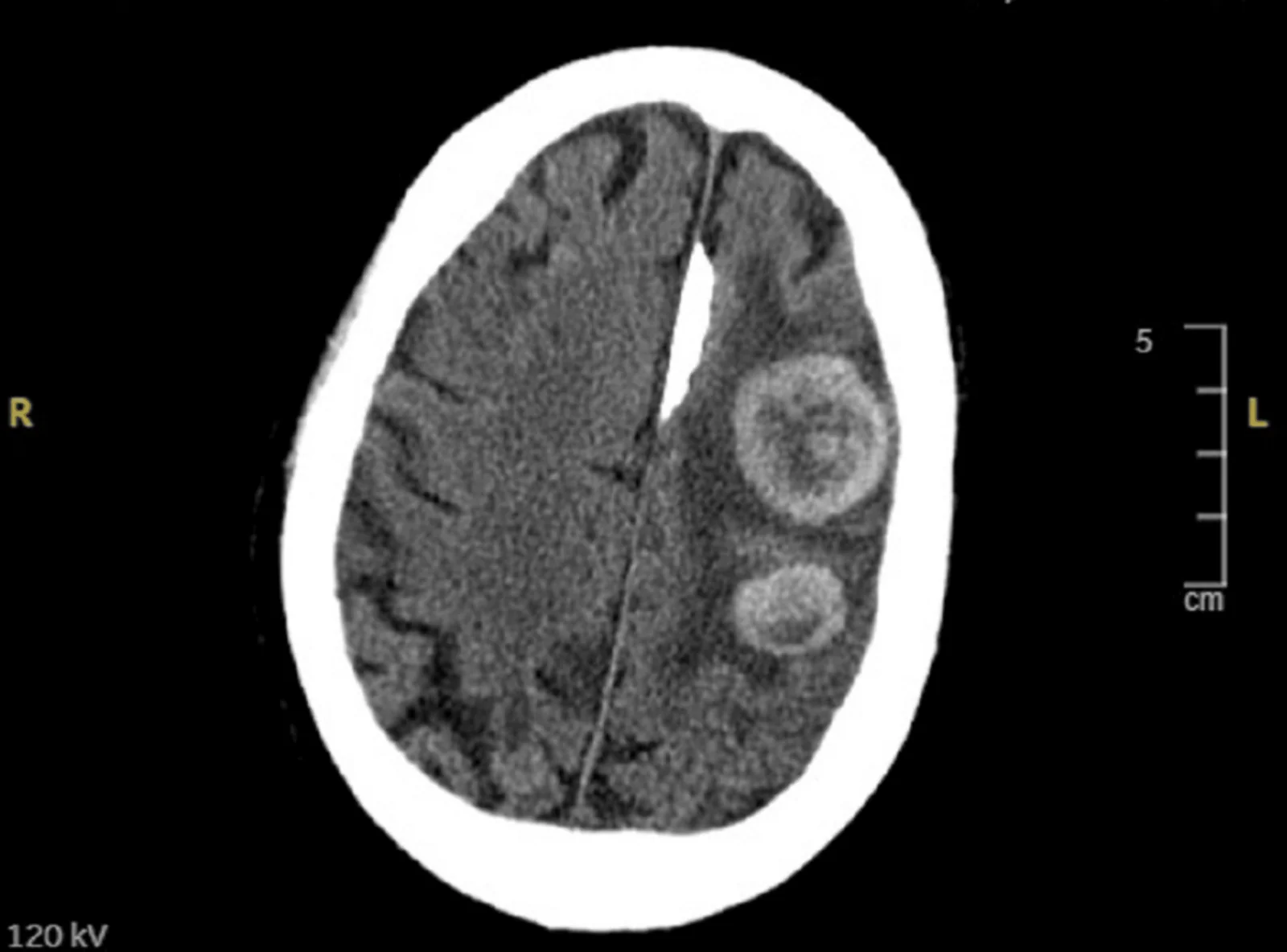
Head CT demonstrating left frontal lobe lesions with associated edema Axial CT of the head demonstrates left frontal lobe intra-axial lesions with associated perifocal edema. The largest lesion measures approximately 2.9 × 3.2 cm CT: computed tomography

Interval imaging demonstrated rapid disease progression, with new subcarinal lymphadenopathy measuring approximately 2.4 × 3.4 cm (Figure 8), bilateral adrenal masses (Figure 9), and a suspicious pancreatic body lesion (Figure 10). Furthermore, head CT demonstrated multiple bilateral lesions previously seen on the outpatient MRI, with the largest lesion visualized within the left frontal lobe and associated with moderate surrounding vasogenic edema (Figure 11).

Before hospitalization, the patient had an Eastern Cooperative Oncology Group (ECOG) performance status of 0 [8]. During hospitalization, however, he experienced rapid clinical deterioration related to progressive metastatic disease. By the time systemic therapy was initiated, his ECOG performance status had declined to 3, reflecting significant functional impairment. Given the patient's advanced-stage disease, rapidly worsening functional status, and poor prognosis, the risks and potential benefits of treatment were discussed with the family, who elected to proceed with systemic therapy. Systemic therapy was ultimately initiated during hospitalization rather than deferred to the outpatient setting. The patient received the first cycle of carboplatin (AUC 6, 700 mg) intravenously over one hour, pemetrexed (500 mg/m², 975 mg) intravenously over 30 minutes, and pembrolizumab (200 mg) intravenously over 30 minutes.

In addition, he underwent five fractions of whole-brain radiotherapy for the treatment of brain metastases, with concurrent initiation of memantine at 5 mg once daily, followed by a planned titration to 5 mg twice daily after seven days, then 10 mg in the morning and 5 mg in the evening for seven days, and subsequently 10 mg twice daily. Memantine was administered concurrently with whole-brain radiotherapy to reduce the risk of radiation-induced cognitive decline, based on evidence from a randomized, double-blind, placebo-controlled trial demonstrating delayed cognitive decline and better preservation of neurocognitive function in patients receiving whole-brain radiotherapy [9]. Dexamethasone was administered intravenously at 6 mg every eight hours for the management of peritumoral cerebral edema, and levetiracetam was administered intravenously at 1,000 mg every 12 hours following a seizure in the setting of intracranial metastatic disease.

**Figure 12:**
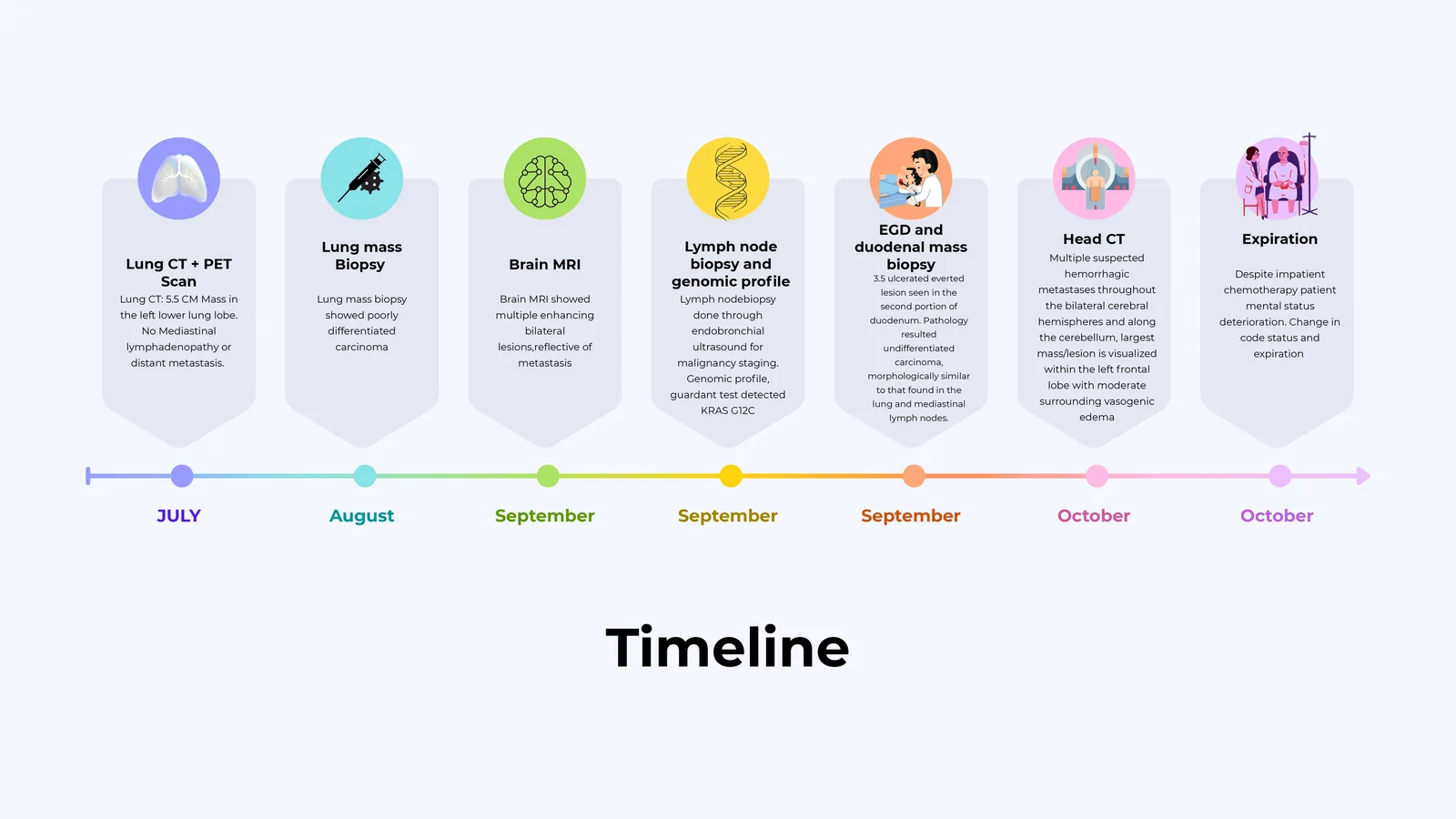
Timeline of diagnostic evaluation, treatment, and clinical outcome Chronological timeline illustrating the major diagnostic investigations, therapeutic interventions, and clinical outcome during the patient’s disease course CT: computed tomography; PET: positron emission tomography; MRI: magnetic resonance imaging; EGD: esophagogastroduodenoscopy The figure was created using Canva®

The patient's hospital course was prolonged, with progressive clinical deterioration in the setting of advanced metastatic disease. He developed worsening lethargy and altered mental status, requiring endotracheal intubation for airway protection and transfer to the intensive care unit. He was subsequently extubated; however, given his overall poor prognosis and continued clinical decline, the family elected to de-escalate his care, and his code status was changed to Do Not Resuscitate/Do Not Intubate (DNR/DNI). The patient ultimately died approximately one week later, three months following the initial diagnosis, as demonstrated in the clinical timeline (Figure 12).

## Discussion

Lung cancer is a prevalent disease; however, GI metastasis is a rare phenomenon, particularly when diagnosed ante mortem. The discrepancy between clinical and post-mortem detection suggests that GI involvement may be underrecognized. Yang et al. [10] reported an incidence of 1.77% of symptomatic GI metastasis among lung cancer patients in a teaching hospital in Taiwan. In contrast, autopsy studies have demonstrated higher rates of GI involvement. Antler et al. reported GI metastases in 14% of patients with lung cancer evaluated at autopsy [11], while Yoshimoto et al. identified GI involvement in 11.9% of autopsied lung cancer cases [12]. Similarly, McNeill et al. described small bowel metastases identified during autopsy evaluation [13]. The small bowel appears to be the most frequently involved GI site. Rossi et al. [6] presented a series of 18 cases of primary lung cancer with GI tract involvement, of which 12 cases involved the small intestine and nine required surgical resection.

The small number of reported cases could be attributed to the fact that endoscopic findings are not specific, ranging from single lesions that mimic a primary GI tumor to diffuse mucosal involvement. Therefore, clinicians and endoscopists should maintain a high index of suspicion when investigating abdominal symptoms and keep a low threshold for obtaining tissue for histologic analysis, which is the most reliable way to identify GI metastasis.

Another relevant aspect for discussion is the mechanism by which lung malignancies metastasize to the GI tract. Although the exact pathways remain incompletely understood, GI metastases from lung cancer are generally believed to occur through hematogenous dissemination, particularly in the setting of advanced disease [14]. The rarity of clinically recognized GI metastases contrasts with findings from autopsy studies, which suggest that GI involvement is more common than previously appreciated and may remain clinically silent until late stages of disease. Further studies are needed to better characterize the molecular mechanisms underlying GI dissemination in lung cancer and to identify patients who may be at increased risk for this uncommon metastatic pattern.

The prognosis is poor, with a median survival of 96.5 days after diagnosis, according to Kim et al. [15]. Chemotherapy is the main treatment and should be directed at the primary cancer. Metastatic GI lesions should be addressed when causing complications such as recurrent bleeding or obstruction. Nemoto et al. [16] concluded that operative management could extend survival and improve quality of life. However, it remains unclear whether aggressive surgical management of digestive tract metastases provides an overall survival benefit. Our case did not require surgical resection, as the lesion was not found to be actively bleeding and did not cause obstruction of the digestive tract. The patient in this case survived only 28 days after GI metastatic lesions were diagnosed.

## Conclusions

Primary lung cancer remains one of the most common malignancies worldwide and a leading cause of cancer-related mortality. Although GI metastases from lung cancer are considered uncommon in clinical practice, their true incidence may be underestimated due to nonspecific clinical manifestations and limited detection during patients' lifetimes. Autopsy studies have demonstrated a higher frequency of GI involvement compared with clinically recognized cases. This report highlights the diagnostic challenge of identifying duodenal metastasis from a poorly differentiated lung carcinoma in the setting of rapidly progressive, widely metastatic disease. Despite the initiation of systemic therapy, the patient experienced rapid clinical deterioration and an unfavorable outcome, reflecting the poor prognosis associated with advanced metastatic disease. Recognition of GI involvement in patients with lung cancer who develop new GI symptoms may assist in appropriate diagnostic evaluation and individualized management decisions; however, extensive metastatic disease may significantly limit therapeutic options and outcomes. Overall, this report emphasizes the importance of integrating clinical presentation, imaging findings, pathology, and molecular profiling to establish the diagnosis and guide management decisions in patients with advanced lung cancer.
